# The Effect of Cardiac Rehabilitation in Paediatric Fontan Circulation Patients: A Prospective Intervention Study

**DOI:** 10.3390/medicina60101566

**Published:** 2024-09-24

**Authors:** Luna van de Ven, Ana Clara Félix, Joana Suarez, Bruno Rodrigues, Jorge Dias, Fátima F. Pinto, Sérgio Laranjo

**Affiliations:** 1 Department of Paediatric Cardiology, Children’s Hospital, University Medical Center Utrecht, P.O. Box 85090, 3508 Utrecht, The Netherlands; 2 Pediatric Cardiology Department, Hospital de Santa Marta, Unidade Local de Saúde São José EPE, 1150-293 Lisbon, Portugal; 3 Clínica Universitária de Cardiologia Pediátrica, Centro Clínico Académico de Lisboa, 1150-293 Lisbon, Portugal; 4 Physical and Medical Rehabilitation Department, Hospital de Santa Marta, Unidade Local de Saúde São José EPE, 1150-293 Lisbon, Portugal; 5 Comprehensive Health Research Center, NOVA Medical School, Faculdade de Ciências Médicas, NMS, FCM, Universidade NOVA de Lisboa, 1169-056 Lisbon, Portugal

**Keywords:** Fontan procedure, cardiac rehabilitation, exercise capacity, health-related quality of life, cardiorespiratory fitness, aerobic exercise training

## Abstract

*Background and Objectives:* The Fontan procedure, a palliative surgery for univentricular heart physiology, often reduces exercise capacity and quality of life. This study aimed to evaluate the impact of cardiac rehabilitation (CR) on improving outcomes in Fontan patients to inform evidence-based care. *Materials and Methods:* Fontan patients aged 8–30 participated in a structured CR program for at least three months. The program included weekly aerobic and resistance training sessions with educational and nutritional guidance. Baseline and post-CR assessments included cardiac function, fitness, daily activity, and health-related quality of life (HRQOL). *Results*: The cohort included ten Fontan patients, of whom six had a right systemic ventricle. CR significantly improved cardiorespiratory fitness, as seen in VO2 max (from 27.92 ± 5.15 to 34.69 ± 1.14 mL/kg/min, *p* = 0.0089) and percent predicted VO2 (from 0.67 ± 0.18 to 0.90 ± 0.02, *p* = 0.005). VCO2 increased by +8.68 ± 8.59 mL/kg/min but did not reach statistical significance (*p* = 0.05). Most haemodynamic and ventilatory parameters showed no significant improvement. All the SF-36 questionnaire domains showed significant HRQOL gains (*p* < 0.001). High adherence (85–93%), no adverse events, and reduced NT-proBNP levels supported the program’s safety. *Conclusions*: This study’s findings have important implications for the care of Fontan patients. CR significantly improved exercise capacity and HRQOL in Fontan patients across various anatomies, particularly in the right systemic ventricle. Integrating physical conditioning into standard care could reduce long-term morbidity and mortality; however, further research is needed to refine the protocols and confirm sustained benefits.

## 1. Introduction

Univentricular heart defects form a complex group of congenital heart diseases (CHDs) that, if untreated, pose a significant mortality risk. The staged Fontan procedure, a crucial palliative intervention, has improved the 30-year survival rate to approximately 85%. However, Fontan circulation leads to long-term complications owing to its passive nature. Venous blood is rerouted directly into the pulmonary arteries, bypassing the right ventricle, so there is no subpulmonary pumping mechanism to increase cardiac output during exertion. This results in exercise intolerance and requires higher central venous pressure to maintain blood flow [1,2,3,4,5,6].

Patients with Fontan circulation face unique physiological challenges that predispose them to complications such as muscle wasting (sarcopenia) and bone demineralisation (osteoporosis), both of which result from reduced physical activity and a lack of weight-bearing exercises [7,8,9]. Elevated venous pressures can also impair cerebral circulation, leading to neurological complications [10], while altered haemodynamics increase the risk of thromboembolic events, further threatening neurological integrity [11]. Additionally, prolonged elevation of central venous pressure predisposes these patients to severe complications, including protein-losing enteropathy and plastic bronchitis. Liver congestion, with subsequent progression to fibrosis or cirrhosis, further complicates the metabolic profile [5,12,13].

Psychosocial burdens are similarly significant, as physical limitations and chronic illness often lead to psychological issues such as low self-esteem, social isolation, and depression [8,9]. Recent evidence suggests that tailored cardiac rehabilitation (CR) programs can help mitigate these multifaceted challenges [8]. These programs typically comprise aerobic training, resistance training, and, in some cases, inspiratory muscle training (IMT). Aerobic and resistance training have been shown to substantially enhance exercise capacity in Fontan patients by improving cardiorespiratory fitness [14,15] and counteracting muscle wasting and bone demineralisation [16,17]. Resistance training of the lower limb muscles may also augment non-pulsatile venous flow, thereby improving venous return and systemic preload [18,19].

Inspiratory muscle training (IMT), which aims to strengthen the respiratory muscles, has gained attention for its potential to enhance pulmonary function in Fontan patients. As these patients rely on passive pulmonary blood flow, IMT can improve the thoracic pump mechanism, increasing venous return, cardiac output, and overall exercise capacity [16]. Beyond the physical benefits, CR programs also promote mental well-being by fostering a sense of community and providing a structured, supportive environment for safe exercise, which can reduce anxiety related to sudden cardiac events [9,20].

This study investigates the effects of a comprehensive CR program, incorporating aerobic, resistance, and inspiratory muscle training, on patients with Fontan circulation. It hypothesises that a holistic rehabilitation approach will improve both physical and psychosocial outcomes, thereby contributing to better long-term management strategies.

## 2. Materials and Methods

### 2.1. Study Design and Participants

This prospective study was conducted at the Paediatric Cardiology Department of the Hospital de Santa Marta, ULS S José, Lisbon, Portugal, with ethical approval from the Medical 2.1. Ethics Committee (CES 1445/2023). The study recruited children and young adults aged 8 to 30 years who had undergone Fontan total cavopulmonary connection (TCPC) palliation for single-ventricle physiology. Participants were enrolled during routine clinic visits at the Congenital Heart Disease clinics and through referrals, ensuring a diverse and representative sample.

Inclusion Criteria:

1. Ages 8 to 30 years.
2. Completion of the Fontan TCPC palliation.
3. Ability to provide informed consent (or consent from guardians of minors).
4. Ability and willingness to participate in a CR program and undergo repeated cardiopulmonary exercise testing (CPET).
5. Impaired aerobic capacity (peak VO2 < 80% predicted).
6. Baseline resting oxygen saturation (SpO2) > 85%.

Exclusion Criteria:

1. Recent (within six months) or planned (within 12 months) cardiac interventions, including catheterisation or surgery.
2. Formal CR program participation in the past 24 months.
3. Current or recent pregnancy (<90 days) or planned pregnancy within the next 12 months.
4. Active heart failure, hospitalisation, or significant clinical changes in the past 30 days.
5. Recent or planned events are likely to affect exercise capacity significantly.
6. Haemodynamically significant lesions (e.g., aortic stenosis), uncontrolled arrhythmias, or other contraindications.
7. NYHA Class IV Heart Failure.
8. Acute illness in the past three months.
9. Active protein-losing enteropathy (albumin < 2.5 mg/dL).
10. Pacemaker dependency.
11. Cognitive delay affecting participation.

Patients who met any of the exclusion criteria were ineligible for the study.

### 2.2. The CR Program

The CR program involved three one-hour weekly training sessions over at least three months. If patients continued to show improvement after this period, they were kept in the program. The sessions were group-based and supervised by a physiotherapist, rehabilitation nurse, and paediatric cardiologist.

In the first three weeks, sessions were conducted in the hospital to familiarise the patients. Subsequently, patients could continue at home with real-time supervision. Each session began with a 10–20 min warm-up of light activities and stretching, followed by 20–40 min of aerobic and resistance training. Aerobic exercises included treadmill walking, cycling, rowing, or dynamic play, while resistance training involved wall pulleys and hand weights. A 10-min cooldown concluded each session.

The training was personalised based on baseline heart rate (HR) and perceived exertion, primarily targeting 60% of the HR reserve. The intensity and duration were increased weekly. Real time, continuous wireless ECG monitor bands assessed each participant’s heart rate and rhythm continuously throughout the sessions. The devices used were capable of continuous ECG monitoring, providing full rhythm strips and enabling the detection of ectopy, arrhythmias, and any significant changes in the ST segment.

Educational sessions led by a rehabilitation nurse covered cardiovascular disease causes, treatment, and medication use. The patients also received guidance on stress reduction, nutrition, smoking cessation, and maintaining a healthy lifestyle.

### 2.3. Clinical Data Collection

The patients’ medical examinations and psychological assessments were performed at baseline and post-intervention. Baseline assessments were performed within two months before CR started, while post-intervention measurements were performed within two weeks after completing the CR program.

All examinations were standard clinical procedures performed using standardised clinical protocols. Physical fitness was assessed using a graded exercise test with an oxygen uptake evaluation. Blood biomarkers were also analysed, focussing on N Terminal-pro Brain natriuretic peptide levels.

### 2.4. Selective Data Presentation Strategy

This study aimed to analyse the effects of CR on Fontan patients by focussing on two key objectives:

- Assessing the impact of the CR program on exercise capacity using cardiopulmonary exercise testing (CPET).
- Assessing the effect of the CR program on health-related quality of life (HRQOL) using the SF-36 questionnaire.

### 2.5. Cardiopulmonary Exercise Testing

The Bruce protocol, a standardised treadmill exercise test, was used for CPET to measure the participants’ maximum effort based on the Borg scale. Respiratory gases and heart rate were continuously monitored to assess key parameters, such as VO2 max, ventilatory anaerobic threshold, heart rate response, and respiratory exchange ratio. These data provide insights into participants’ aerobic capacity and cardiovascular efficiency.

### 2.6. Health-Related Quality of Life Assessments

The SF-36 questionnaire was used to evaluate the impact of CR on patients’ health-related quality of life (HRQOL) across domains like physical functioning, general health, vitality, social functioning, and mental health [21]. Semi-structured interviews conducted by a rehabilitation nurse offered a comprehensive assessment of the effects of the CR program on HRQOL, emotional functioning, social participation, health-related behaviours, leisure activities, and disease-specific knowledge.

### 2.7. Statistical Data Analysis

Python (version 3.8) was used for the data analysis and visualisation. Key libraries included Pandas (1.1.3) for data management, NumPy (1.19.2) for computations, SciPy (1.5.2) for statistical tests, and Matplotlib (3.3.2) with Seaborn (0.11.0) for plots. All analyses were performed in a Jupyter Notebook hosted on Google Colab for transparency and replicability.

The Shapiro–Wilk test was used to check data normality, guiding the choice of statistical tests. Paired t-tests were used for normally distributed data, and the Wilcoxon signed-rank test was used for non-normally distributed data. A significance level of *p* < 0.05 was used for all analyses.

Continuous variables are reported as the mean ± standard deviation. Categorical variables were analysed for frequency distributions to describe the study population and adherence rates. Adherence was calculated as the percentage of attended sessions of the total number prescribed.

## 3. Results

### 3.1. Patient Demographics and Baseline Clinical Characteristics

The study involved ten patients who had undergone Fontan TCPC palliation, including six females, with an average age of 15.6 ± 1.7 years. The most common anatomy was hypoplastic left heart syndrome, which was seen in four patients. Other conditions included tricuspid atresia and double-outlet right ventricle (two patients each), while one patient each had an unbalanced atrioventricular canal, pulmonary atresia, and complex congenital anatomy. One patient was diagnosed with heterotaxy syndrome; however, no other genetic or systemic syndromes were identified (Table 1).

The Fontan TCPC palliation was performed on patients with an average age of 4.8 ± 0.8 years and an average weight of 20.7 ± 2.4 kg. Seven patients underwent extracardiac conduit surgery, and three underwent a lateral tunnel procedure, all with fenestration during surgery. None of the patients had phrenic nerve paresis, leading to diaphragmatic paresis.

Six patients had a right systemic ventricle, and four had a left systemic ventricle. Recent echocardiograms showed nine patients with normal to mildly reduced ventricular function and one with moderately reduced function. Eight patients had no to mild atrioventricular valve regurgitation, while two had moderate regurgitation. None of the patients had mild aortic or neo-aortic regurgitation. Resting saturation averaged 90 ± 3%, dropping to 86 ± 2% at peak exercise.

The mean TCPC pressure at the latest catheterisation was 15.9 ± 1.8 mmHg, although some tests were conducted 2–3 years before starting the CR program. At the start of CR, three participants were already receiving bosentan, and one patient was receiving sildenafil due to evidence of elevated pressures within the Fontan circuit. This medication, along with others, was part of their standard treatment regimen prior to enrolling in the rehabilitation program. All patients received anticoagulants and diuretics according to the protocol. Two patients with significant Fontan dysfunction were listed for heart transplantation before CR.

The patients attended an average of 69 exercise sessions (range 25–105). Pre- and post-intervention assessments were spaced by 32.1 ± 7.7 weeks, with an average interval of 11.7 ± 3.4 years between TCPC palliation and the start of CR.

### 3.2. The Effect of CR in Fontan Patients

#### 3.2.1. Enhancement of Cardiorespiratory Fitness

**Figure 1:**
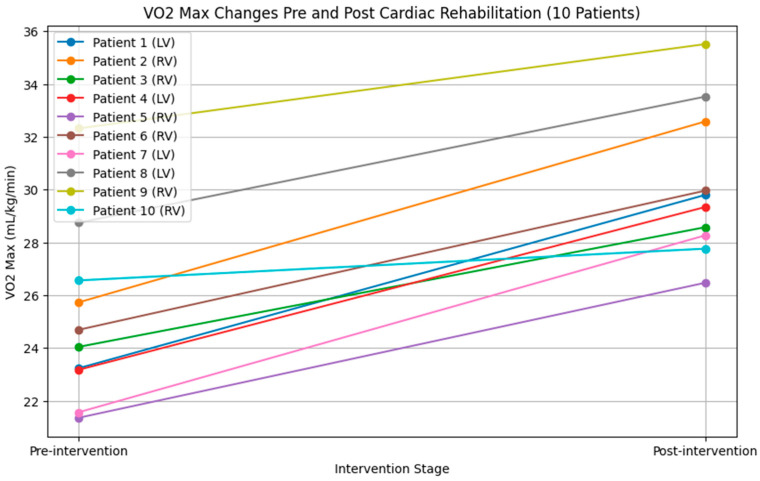
The effect of Cardiac Rehabilitation (CR) on aerobic capacity. This figure illustrates the variation in VO2 Max of the ten Fontan patients before and after participating in the CR program. The plot captures individual trajectories of change in aerobic capacity, as evidenced by the VO2 Max measurements. All patients showed improved VO2 Max after participating in the program. Abbreviations: LV = left systemic ventricle Fontan patient. RV = right systemic ventricle Fontan patient. VO2 max = maximum oxygen consumption.

The VO2 max was used to evaluate aerobic capacity, with an initial average of 27.92 ± 5.15 mL/kg/min, which increased to 34.69 ± 1.14 mL/kg/min post-intervention—a significant rise of 6.77 ± 6.35 mL/kg/min (*p* = 0.0089). Predicted VO2 also showed significant improvement, increasing from 0.67 ± 0.18 to 0.90 ± 0.02 (mean change: 0.23 ± 0.17, *p* = 0.005). Metabolic efficiency, measured via VCO2, rose from 31.65 ± 6.68 mL/kg/min to 40.33 ± 1.42 mL/kg/min post-CR, but this change of 8.68 ± 8.59 mL/kg/min was not statistically significant (*p* = 0.05). The results are shown in Figure 1 and Table 2.

#### 3.2.2. Haemodynamic Parameters

Peak oxygen saturation levels improved significantly after cardiac rehabilitation, increasing from 86% ± 2 pre-CR to 92% ± 6 post-CR (mean change: 6% ± 7, *p* = 0.035). Resting oxygen saturation remained unchanged. No significant changes occurred in resting or peak heart rates post-CR. The resting systolic blood pressure increased from 107 ± 5 mmHg to 117 ± 12 mmHg (mean change: 10 ± 13 mmHg, *p* = 0.102), and the peak systolic pressure increased from 139 ± 6 mmHg to 148 ± 9 mmHg (mean change: 9 ± 11 mmHg, *p* = 0.067), but the difference was not statistically significant. The results are presented in Table 3.

Echocardiograms were conducted at both the start and conclusion of the cardiac rehabilitation program. No significant changes in ventricular function were observed between the pre- and post-CR assessments.

#### 3.2.3. Ventilatory Parameters

The tidal volume increased significantly from 1.4 ± 0.7 L to 2.5 ± 0.7 L (*p* = 0.014) post-CR. No significant changes were observed in other ventilatory measures (Table 4). The maximum respiratory rate increased from 55.3 breaths/min to 63.2 breaths/min without reaching statistical significance (*p* = 0.264). Likewise, the maximum minute ventilation and ventilatory efficiency ratios VE/VO2, VE/VCO2, and respiratory exchange ratio showed no significant changes.

#### 3.2.4. Enhancement of HRQOL

The SF-36 questionnaire showed significant improvements across key areas (*p* < 0.001) (Table 5). Physical and social functioning scores increased, similar to the general health perceptions. Vitality scores, which reflect energy and fatigue, also improved substantially. Mental health gains were noted through reduced distress and enhanced well-being.

Semi-structured interviews supported these results, with several patients starting or returning to work. Notably, the two patients awaiting heart transplantation experienced marked improvement, leading to a re-evaluation of their transplant status.

### 3.3. Adherence and Safety of the CR Program

Patients attended 85–93% of the CR training sessions. No adverse events occurred, and no patients withdrew from the study. Mean NT pro-BNP levels decreased by 14.2 pg/mL after CR (from 192.4 ± 63.6 pg/mL pre-CR to 178.2 ± 5.5 pg/mL post-CR).

## 4. Discussion

This study provides new insights into the application of a combined aerobic exercise (AET) and resistance training program in Fontan patients, a population with significant physiological challenges and heterogeneous anatomical subtypes. While previous studies have explored the effects of AET alone or in combination with inspiratory muscle training (IMT), our study is one of the few to focus on the impact of integrating resistance training with AET. This approach not only led to a substantial improvement in exercise capacity, particularly in patients with lower baseline fitness, but also demonstrated significant enhancements in health-related quality of life (HRQoL). Furthermore, the inclusion of a cohort with a high proportion of patients with right systemic ventricles (60%)—a group typically associated with worse exercise outcomes—underscores the potential of this tailored CR program to benefit a broader range of Fontan patients. The safety and efficacy of this program, without adverse events, also highlight its viability as a rehabilitation strategy for this vulnerable population.

### 4.1. The Effect of CR on Exercise Capacity

This study aimed to evaluate the impact of a cardiac rehabilitation (CR) program on the exercise capacity of Fontan patients. Exercise tolerance depends on pulmonary gas exchange, skeletal muscle metabolism, and cardiovascular function; therefore, assessing VO2 max, VCO2, and minute ventilation are crucial to gauge exercise capacity [22].

VO2 max, the gold standard for evaluating aerobic fitness [23,24], showed a significant increase of +6.77 ± 6.35 mL/kg/min, reflecting improved endurance and exertion capacity. Percent predicted VO2 also rose by 0.23 ± 0.17 (34.3%), reaching a post-CR mean of 0.90 ± 0.02. This 34.3% improvement in oxygen absorption brought participants close to normative levels, as values under 85% were considered abnormal [25,26].

VCO2 also increased by +8.68 ± 8.59 mL/kg/min post-CR, though it narrowly missed statistical significance (*p* = 0.050). This enhanced metabolic response points to improved handling of higher exercise intensities. Apart from tidal volume, ventilatory efficiency also improved, although most measures remained unchanged.

Overall, the remarkable increase in percent predicted VO2 signifies a substantial improvement in exercise capacity, enabling patients to engage in more varied activities and leading to a healthier lifestyle.

These results are consistent with those in the existing literature. A systematic review involving 264 Fontan patients showed a modest 1.7 mL/kg/min (6%) increase in VO2 max [8]. However, this study’s greater improvement is likely due to a lower baseline VO2 max of 27.92 ± 5.15 mL/kg/min. Similarly, Ali et al. found a significant increase post-CR with a low baseline value [27]. In contrast, Duppen et al. found no significant improvement with a high baseline VO2 max of 33 mL/kg/min [28]. Thus, patients with a lower initial VO2 max gain the most from a CR.

While patients with a right systemic ventricle often face worse exercise capacity than those with a left systemic ventricle [29,30,31], 60% of the cohort in this study had a right systemic ventricle. Despite this challenge, the results prove that patients with the right systemic ventricles can increase their exercise capacity through rehabilitation and benefit from CR.

### 4.2. The Effect of CR on HRQOL

The SF-36 questionnaire and semi-structured interviews revealed a significant improvement in Fontan patients’ health-related quality of life (HRQoL) post-CR. The results showed physical, emotional, and psychological health improvements, including better management of chronic fatigue, increased social engagement, and greater self-confidence and independence. Overall, this results in a more positive health outlook. These results align with previous studies that reported improvements in HRQoL post-CR [15,28,32].

However, these studies found that enhanced exercise capacity does not always correlate with an improved HRQoL. Dirks et al. noted better exercise capacity via VO2 max measurements, yet HRQoL remained unchanged [16]. In contrast, Duppen et al. and Hedlund et al. reported improved HRQoL without significant gains in VO2 max post-CR [28,32]. This suggests that, while exercise capacity is important, other aspects of CR also significantly impact the HRQoL of Fontan patients.

### 4.3. Optimisation of CR for Fontan Patients

This study’s CR program combined AET with resistance training, resulting in improved exercise capacity in Fontan patients. The enhancement came from gains in cardiac and muscular parameters, whereas most ventilatory parameters showed no significant improvement. Pulmonary function is crucial for Fontan patients because pulmonary blood flow is not actively propelled [5]. Research shows that inspiratory muscle training (IMT) can improve pulmonary function by enhancing the thoracic pump and increasing blood flow, preload, cardiac output, and exercise capacity [16].

Abdulkarim et al. suggested that combining AET with IMT could improve exercise capacity and pulmonary function in Fontan patients [13]. However, resistance training also appears promising, as Scheffers et al. found that leg-focused high-weight resistance training significantly improved exercise capacity after 12 weeks [33]. The optimal combination of training remains debated; however, AET is fundamental, and pairing it with resistance training seems promising. Adding IMT may be valuable for enhancing ventilatory parameters.

The absence of improvement in ventilatory parameters may be due to the short three-month duration of this study. Pulmonary issues such as restrictive lung diseases, plastic bronchitis, and cyanosis are common in 61% of patients with TCPC [34]; therefore, meaningful improvements may require a longer rehabilitation period.

### 4.4. Safety of CR for Fontan Patients

The CR program was evaluated as safe, consistent with the current literature [8]. However, it should be mentioned that this study did not include patients with a failing Fontan circulation. Therefore, no statements could be made regarding the safety of exercise training for this type of patient.

### 4.5. Study Limitations

This study has several limitations. Without a Fontan control group, whether the observed improvements were directly due to the CR program or whether a learning curve influenced assessment performance is unclear. Additionally, the study’s small cohort limited the statistical power, and while the diverse population reflects the heterogeneity of Fontan patients, a larger group is needed for better statistical analysis.

Cardiac function was mainly assessed through echocardiography owing to logistical and clinical constraints, despite cardiac MRI being the gold standard. Although cardiac MRI was used in some cases, differences between the imaging modalities could have led to a conservative estimate of cardiac function.

The CR program lasted at least three months, with participants completing between 25 and 105 sessions. Some patients stayed in the program longer if they showed continued improvement. However, the small cohort size made conducting a sub-analysis of the CR duration difficult. Thus, no conclusion can be drawn regarding the optimal CR duration for Fontan patients.

### 4.6. Future Research

Our study was limited by the small sample size and a follow-up period of just three months. Future research should investigate this CR program with a larger cohort and longer follow-up, ideally over at least a year, as the long-term effects of CR in Fontan patients remain unexplored. This study found improved exercise capacity due to improvements in cardiac and muscle parameters. Investigating whether ventilatory parameters improve over a longer period of time would be valuable.

There is no consensus regarding the optimal CR program for Fontan patients. Although some studies suggest that combining AET and IMT is the most effective, this study evaluated AET with resistance training. Further research is needed to identify the most beneficial combination for Fontan patients.

## 5. Conclusions

This study examined the impact of the CR program on exercise capacity and HRQOL in Fontan patients. The results showed improved exercise capacity and HRQOL, even with 60% of the study population having the right systemic ventricle. This underscores the importance of physical conditioning for enhancing the quality of life of patients with Fontan circulation. We recommend that all Fontan patients engage in regular exercise, and further research is needed to optimise CR to reduce morbidity and mortality in this population.

## Figures and Tables

**Table 1:** Summary of Patient Demographics and Baseline Clinical Characteristics Data.

Variable	Value
Age at start of study (years)	15.6 ± 1.7
Gender (female)	6 (60%)
Syndrome	1 (10%)
Comorbidities	0 (0%)
Anatomical variations	
Hypoplastic left heart syndrome	4 (40%)
Tricuspid atresia	2 (20%)
Double outlet right ventricle	2 (20%)
Unbalanced atrioventricular canal hypoplastic left ventricle	1 (10%)
Pulmonary atresia	1 (10%)
Anomaly of pulmonary vein return, double inlet, double outlet	1 (10%)
Fontan TCPC palliation	
Age at TCPC palliation (years)	4.8 ± 0.8
Weight at TCPC palliation (kg)	20.7 ± 2.4
Time interval between TCPC palliation and CR (years)	11.7 ± 3.4
Extra-cardiac conduit	7 (70%)
Lateral tunnel	3 (30%)
Fenestration present at initial operation	10 (100%)
Fenestration open at start of CR	9 (90%)
LPA stent	1 (10%)
Diaphragm paresis	0 (100%)
Fontan function	
TCPC pressure at latest heart catheterisation (mmHg)	15.9 ± 1.8
SaO_2_ rest (%)	90 ± 3
SaO_2_ peak exercise (%)	86 ± 2
Fontan circulation disfunction	2 (20%)
Medication	
Bosentan	3 (30%)
Sildenafil	1 (10%)
Enalapril	4 (40%)
Ventricular function	
Right systemic ventricle	6 (60%)
Left systemic ventricle	4 (40%)
Normal to mildly diminished ventricular function	9 (90%)
Moderately diminished ventricular function	1 (10%)
None to mild atrioventricular valve regurgitation	8 (80%)
Moderate atrioventricular valve regurgitation	2 (20%)
None to mild aortic or neo-aortic regurgitation	10 (100%)
CR program	
Number of exercise sessions attended	69 (25–105)
Time between pre- and post-intervention testing (weeks)	32.1 ± 7.7

**Table 2:** Effect of CR on Cardiorespiratory Fitness.

Variable	N	Pre-CR (Mean ± SD)	Post-CR (Mean ± SD)	Change Score (Mean ± SD)	*p*-Value
VO2 Max (mL/kg/min)	10	27.92 ± 5.15	34.69 ± 1.14	6.77 ± 6.35	0.009 *
Percent predicted VO2	10	0.67 ± 0.18	0.90 ± 0.02	0.23 ± 0.17	0.005 *
VCO2 (mL/kg/min)	10	31.65 ± 6.68	40.33 ± 1.42	8.68 ± 8.59	0.050

Abbreviations: N = number of included patients. CR = cardiac rehabilitation. VO2 max = maximum oxygen consumption. VCO2 = carbon dioxide production. * = indicates significant findings, *p* < 0.05.

**Table 3:** Effect of CR on Haemodynamic Parameters at Rest and Peak Exercise.

Variable	N	Pre-CR (Mean ± SD)	Post-CR (Mean ± SD)	Change Score (Mean ± SD)	*p*-Value
Resting HR (bpm)	10	91 ± 10	88 ± 8	−3 ± 13	0.619
Peak HR (bpm)	10	175 ± 7	177 ± 10	2 ± 12	0.590
Resting Systolic BP (mmHg)	10	107 ± 5	117 ± 12	10 ± 13	0.102
Peak Systolic BP (mmHg)	10	139 ± 6	148 ± 9	9 ± 11	0.067
Resting Diastolic BP (mmHg)	10	67 ± 6	61 ± 4	−6 ± 8	0.090
Peak Diastolic BP (mmHg)	10	58 ± 8	63 ± 10	5 ± 13	0.312
Resting SaO_2_ (%)	10	90 ± 3	93 ± 2	3 ± 3	0.064
Peak SaO_2_ (%)	10	86 ± 2	92 ± 6	6 ± 7	0.035 *

Abbreviations: N = number of included patients. CR = cardiac rehabilitation. HR = heart rate. BP = blood pressure. SaO_2_ = oxygen saturation. * = indicates significant finding, *p* < 0.05.

**Table 4:** Effect of CR on Ventilatory Measures at Peak Exercise.

Variable	N	Pre-CR (Mean ± SD)	Post-CR (Mean ± SD)	Change Score (Mean ± SD)	*p*-Value
Maximum Minute Ventilation (L/min)	10	91.6 ± 28.4	92.2 ± 26.5	0.6 ± 37.0	0.766
TV (L)	10	1.4 ± 0.7	2.5 ± 0.7	1.1 ± 1.0	0.014 *
Maximum Respiratory Rate (breaths/min)	10	55.3 ± 14.2	63.2 ± 11.3	7.9 ± 18.1	0.264
VE/VO2	10	34.3 ± 5.2	32.2 ± 5.1	−2.1 ± 7.3	0.509
VE/VCO2	10	34.7 ± 3.1	35.8 ± 2.1	1.1 ± 3.7	0.534
Respiratory Exchange Ratio	10	1.19 ± 0.02	1.12 ± 0.02	−0.07 ± 0.02	0.782

Abbreviations: N = number of included patients. CR = cardiac rehabilitation. TV = tidal volume. VE = minute ventilation. VO2 = oxygen consumption. VCO2 = carbon dioxide production. * = indicates significant findings, *p* < 0.05.

**Table 5:** Effect of CR on HRQOL Outcomes.

HRQOL Domain	N	Pre-CR (Mean ± SD)	Post-CR (Mean ± SD)	Change Score (Mean ± SD)	*p*-Value
Physical Functioning	10	40.3 ± 10.0	61.5 ± 10.5	21.2 ± 14.5	<0.001 *
General Health Perceptions	10	45.2 ± 10.0	65.8 ± 10.5	20.6 ± 14.5	<0.001 *
Vitality (Energy/Fatigue)	10	35.5 ± 12.0	57.1 ± 12.6	21.6 ± 17.4	<0.001 *
Social Functioning	10	50.7 ± 18.0	75.7 ± 18.9	25.0 ± 26.1	<0.001 *
Mental Health	10	42.1 ± 10.0	67.2 ± 10.5	25.1 ± 14.5	<0.001 *

Abbreviations: HRQOL = health-related quality of life. N = number of included patients. CR = cardiac rehabilitation. * = indicates significant findings, *p* < 0.05.

## Data Availability

The data supporting the reported results in this study are not publicly available due to privacy and ethical restrictions. The study involved patient medical records, and the data were anonymised to protect patient confidentiality in accordance with institutional guidelines. Access to the data are restricted and may be available from the corresponding author upon reasonable request, subject to approval by the Institutional Review Board and in compliance with applicable data protection regulations.
